# Characterization and Differentiation of Flavor Profile of 12 Air-Dried Yak Meat Products Using GC–IMS and Multivariate Analysis

**DOI:** 10.3390/foods13162626

**Published:** 2024-08-21

**Authors:** Qiuyu Wang, Rongsheng Du, Yuqi Wang, Shulin Zhang, Linlin Wang, Lina Wang

**Affiliations:** 1 College of Food Science and Technology, Southwest Minzu University, Chengdu 610041, China; akkko_858@163.com (Q.W.); wyq010728@hotmail.com (Y.W.); 17371823406@163.com (S.Z.); 2 Sichuan Institute of Musk Deer Breeding, Chengdu 611800, China; durs05@163.com; 3 School of Food and Biological Engineering, Hefei University of Technology, Hefei 230601, China; 4 Lu’an Soyea Electrical Manufactring Co., Ltd., Lu’an 237000, China

**Keywords:** air-dried yak meat, volatile organic compounds, GC–IMS, PLS-DA, fingerprint

## Abstract

Volatile organic compounds (VOCs) in food are key factors constituting their unique flavor, while the characteristics of VOCs in air-dried yak meat (AYM) from various regions of the Tibetan Plateau and their inter-regional differences remain unclear. Therefore, this study conducted a comprehensive analysis of VOCs in the five-spice (FS), spicy and numbing (SN), and aromatic and spicy (AS) versions of AYM from four regions of the Tibetan Plateau (Gansu, Qinghai, Sichuan, and Tibet) using gas chromatography–ion mobility spectrometry (GC–IMS) A total of 58 VOCs were identified, with alcohols accounting for 28.40%, ketones 22.89%, aldehydes 18.85%, and terpenes 17.61%. Topographic plots, fingerprint profiles, and multivariate analysis not only distinguished AYM of the same flavor from different regions but also discriminated those of different flavors within the same region. Furthermore, 17 key VOCs were selected as the primary aroma characteristics of the 12 types of AYM, including linalool, 3-methylbutanal, acetone, and limonene. Meanwhile, the differential VOCs for each flavor were determined, with linalyl acetate being unique to the FS, (E)-ocimene and ethyl propanoate being specific to the SN, and 2-methyl-3-(methylthio)furan-D and Hexanal-D being characteristic of the AS flavor. Based on the above results, the flavor of AYM can be improved to suit the taste of most people and increase its consumption.

## 1. Introduction

Yak is an important ecological species that can adapt to the harsh alpine and hypoxic climate environment of the Qinghai-Tibet Plateau [1]. The protein content of yak meat ranges from approximately 21.16% to 22.91%, which is slightly higher than ordinary beef and significantly higher than that of pork or chicken. Its essential-amino-acid-to-total-amino-acid ratio ranges from 39.76% to 41.66%, closely approximating the recommended value of 40% set by the Food and Agriculture Organization and the World Health Organization, demonstrating superior nutritional balance. Additionally, yak meat boasts a low fat content of merely 1.54% to 2.43%, far below the 10% to 20% range commonly found in other domestic livestock, making it a healthy meat option characterized by its high-protein and low-fat profile [2]. However, the sales volume of yak meat in daily life is significantly lower than that of other meats, primarily attributed to the fact that yaks are predominantly distributed in the Qinghai-Tibet Plateau region. The storage conditions for fresh yak meat are extremely demanding, leading to high transportation costs, prompting herders to often process it into air-dried yak meat (AYM) [3]. AYM is a highly distinctive traditional raw meat product in pastoral areas, primarily produced by herders utilizing natural air-drying conditions during their long-term nomadic life. It is made from fresh yak meat, which is segmented, sliced, and naturally air-dried under low-temperature and dry conditions, resulting in a unique taste and rich flavor [4]. There are many flavors of AYM, including five-spice (FS), spicy and numbing (SN), aromatic and spicy (AS), and original. Simultaneously, the dried yak beef produced in different regions of the Qinghai-Tibet Plateau exhibits variations in nutritional composition, flavor characteristics, and sensory qualities due to factors such as natural environmental conditions, processing techniques, and operational key points [5,6].

Currently, in the existing research reports on air-dried meat, Ma et al. [3] linked lipid oxidation to protein oxidation and reduced digestibility in traditional AYM processing. Meanwhile, Jia et al. [7] explored NaCl alternatives for dry-cured meat to reduce sodium while maintaining quality. On another front, Fan et al. [8] found microwave drying optimal for yak meat jerky quality, enhancing color, sensory attributes, and amino acid content. Moreover, Kim et al. [9] demonstrated that a collagen–konjac blend improves duck meat jerky quality. In addition, Han et al. [10] analyzed the effects of altitude and drying time on Tibetan yak beef jerky, emphasizing volatile organic compounds’ (VOCs) enhancement and consumer preference. However, the existing studies have primarily focused on exploring the factors affecting the quality of air-dried meat from the perspective of processing techniques. There is a lack of clarity in understanding the differences in VOCs present in air-dried meat from different regions and flavors, particularly in the case of AYM, and the specific variations among these VOCs.

Gas chromatography–ion mobility spectrometry (GC–IMS) is an emerging analytical technique that boasts high separation efficiency, resolution, and sensitivity [11]. Compared to gas chromatography–mass spectrometry (GC–MS), GC–IMS exhibits superior temporal resolution, enabling the online monitoring of dynamic processes, such as the tracking of volatile evolution during thermal treatments, food fermentation, or the release of volatiles during food consumption [12]. In the realm of meat research, GC–IMS has been successfully utilized to characterize VOCs and flavor profiles of Jingyuan lambs at various ages [13], analyze the volatile constituents contributing to flavor development during the production of Dezhou braised chicken [14], and conduct a thorough and systematic investigation of flavor characteristics across different yak muscle types [15]. From the aforementioned studies, it is apparent that GC–IMS plays a pivotal role in the detection and identification of air-dried meat. However, to date, there is a lack of reports investigating the applicability of this technology in identifying VOCs in AYM.

This study utilized GC–IMS to analyze and identify the VOCs in three flavors (FS, SN, and AS) of AYM sourced from four regions of the Qinghai-Tibet Plateau (Gansu, Qinghai, Sichuan, and Tibet). To date, there is no published literature investigating whether there are variations in the VOCs of AYM with the same flavor across these four regions. Considering the pronounced differences in altitude and climatic conditions among the four regions of the Qinghai-Tibet Plateau, it is conceivable that these factors may influence the quality of yak meat, which is the primary ingredient in AYM. We further conducted a differential analysis of VOCs in AYM of the same flavor from these four regions using PCA (principal component analysis) and PLS-DA (Partial Least Squares-Discriminant Analysis). The objective is to identify the VOC profiles of 12 types of AYM and explore the differential flavor compounds among AYM of the same flavor type from different regions, as well as the reasons for taste differences among different flavors within the same region. Ultimately, we aim to provide a theoretical foundation for the identification and flavor enhancement of AYM.

## 2. Materials and Methods

### 2.1. Samples

Twelve varieties of AYM from four regions of the Tibetan Plateau were purchased on the Internet, of which the same three flavors (FS, SN, and AS) of AYM were purchased as test materials from each region. Furthermore, all purchased experimental materials were produced and sold by food companies in the selected regions, and their production dates were similar. Sample information is provided in Table 1.

### 2.2. GC–IMS Analysis

VOCs in AYM samples were identified using a GC–IMS system (FlavourSpec^®^, G.A.S. Instrument, Dortmund, Germany) equipped with an MXT-5 capillary column (15 m × 0.53 mm × 1 μm). Accurately weighed 2 g samples were placed in 20 mL headspace vials and incubated at 40 °C for 15 min. Subsequently, a 500 µL aliquot of headspace gas was automatically withdrawn using an 85 °C syringe and injected into the GC injector, while the column temperature was maintained at 60 °C. Nitrogen gas with a purity of 99.999% was used as both the carrier and drift gas. The IMS temperature was set at 45 °C, and the drift gas flow rate was fixed at 150 mL/min, with a total run time of 20 min. The carrier gas flow rate was initially set at 2 mL/min for the first 2 min, then increased to 100 mL/min and maintained until the end of the analysis. Triplicate experiments were conducted for each sample to ensure consistency and reproducibility. Using the Vocal software for data collection and analysis, the application incorporates the Reporter and Gallery Plot plugins to generate two-dimensional and fingerprint chromatograms of volatile components. With n-ketones C4~C9 as external standards, the retention indices (RI) of volatile components are calculated and then compared with the comprehensive database of the National Institute of Standards and Technology (NIST) and the IMS database for qualitative analysis.

### 2.3. Statistical Analysis

The peak area normalization method was utilized to calculate the relative content of each VOC. Multivariate statistical analyses such as PCA, PLS-DA, and significance analyses of variable projections were performed using SIMCA 14.1. Cluster heatmap analysis was conducted using Origin 2022 software.

## 3. Results

### 3.1. GC–IMS Topography of AYM with Different Flavors in Four Regions

**Figure 1:**
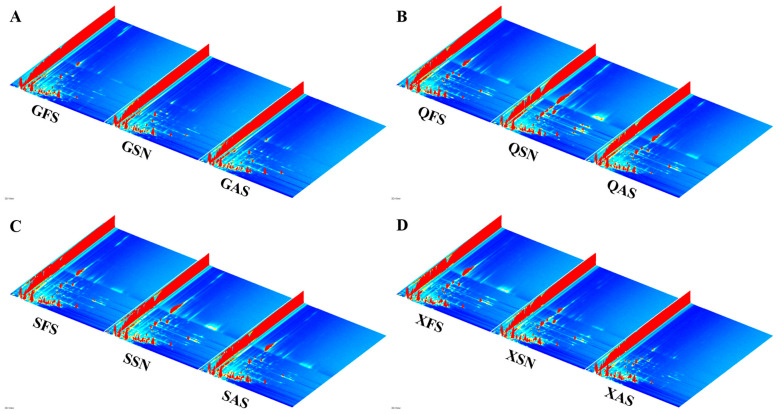
3D topography of air-dried yak meat with different flavors from four regions ((**A**–**D**) are Gansu, Qinghai, Tibet, and Sichuan regions, respectively).

Figure 1 illustrates the 3D plots of AYM with different flavors and origins created by the Reporter plugin. The four 3D plots, arranged from left to right, represent the FS, SN, and AS flavors. The *x*-axis, *y*-axis, and *z*-axis represent the drift time of ion compounds, the retention time of gas chromatography, and the peak signal intensity, respectively. The variations in peak signal intensity between each sample indicate diversity in the VOCs’ content.

**Figure 2:**
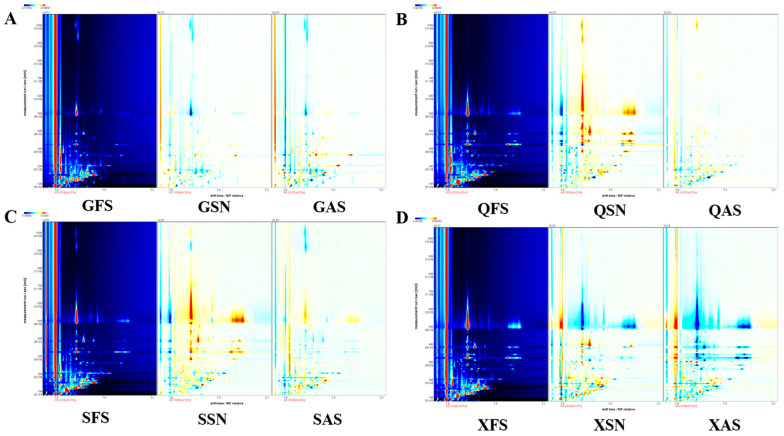
Two-dimensional spectrograms of air-dried yak meat with different flavors from four regions ((**A**–**D**) are Gansu, Qinghai, Tibet, and Sichuan regions, respectively).

To more intuitively compare the differences in VOCs of AYM with various flavors from four different origins, a differential plot of VOCs is constructed, as shown in Figure 2A–D. Taking the FS plot of each origin as a reference, the red vertical line at the abscissa of 1.0 represents the reaction ion peak. Each point to the right of the reaction ion peak represents a VOC, and different colors represent the concentration of the substance, with deeper red indicating a higher concentration and white indicating a lower concentration. The response signals of the remaining sample plots that are identical to the FS of the corresponding origin are subtracted to obtain the differential plot between the two. White color after background subtraction indicates that the concentrations of the two flavor substances are the same, red indicates that the concentration of that substance is higher than the control, and blue indicates that it is lower. The deeper the color, the greater the difference.

The results indicate that there are multiple signal peaks present between the retention times of 100 to 800 s and drift times ranging from 1.0 to 1.7 s. Some differences in the three flavors of Gansu can be seen in Figure 2A. As shown in Figure 2B,C, compared to the FS samples, the SN samples from Qinghai and Sichuan exhibit more red points within the retention time range of 300–700 s, suggesting a higher VOC signal peak. In contrast, SN and AS from Tibet show more blue points (Figure 2D), indicating a reduced VOC signal peak in these two samples. The variations in signal peak intensities for the same flavor across diverse regions are likely attributed to variations in the processing techniques and key technological points employed by herders living in different areas for the production of AYM.

It can be concluded from the above that the VOC content in the three flavors from Gansu province differs, but the difference is not significant. QFS has a lower VOC content than QSN and is almost the same as QAS. SSN and SAS have higher VOC content than SFS, and SSN has more than SAS. Most of the VOC content in XSN and XAS is less than that in XFS.

### 3.2. Identification of VOCs in AYM

A total of 75 signal peaks were detected in 12 samples of AYM, and 58 VOCs (monomers and dimers were counted only once) were identified. The formation of dimers is related to the high proton affinity of the analytes. Compounds with proton affinity higher than water will be ionized and form dimers or polymers [16]. As shown in Table 2, the VOCs include 13 terpenes, 11 aldehydes, 11 esters, 9 alcohols, 5 ketones, 3 acids, and 6 other compounds.

The results showed that the VOCs in AYM were mainly composed of terpenes, aldehydes, and esters, which were roughly consistent with previous findings from studies on yak meat, roasted mutton skewers, and instant sea cucumbers. [17,18,19]. However, among the 12 types of AYM, linalool was found to be the most abundant compound, followed by acetone, β-pinene, and 2-methylbutanal. Linalool, which is recognized as the primary VOC in the pericarp of Zanthoxylum schinifolium [20], possesses a pungent taste and rich aroma, and is used as a pickling ingredient in the food industry [21]. Meanwhile, limonene and linalool are the main flavor components of star anise volatile oil [22]. In the production of AYM, star anise volatile oil is added to the raw meat, enhancing its flavor while also serving the functions of antibacterial, deodorizing, and preservative effects. This finding aligns with the research conducted by Li et al. [19] on instant sea cucumbers, indicating that a substantial amount of spices with antibacterial and preservative properties may be incorporated into instant food products to ensure food quality against deterioration. Acetone primarily contributes to the pungent, irritant, and vinegar-like aroma of food, which may originate from the oxidation of proteins and lipids during the natural air-drying process of yak meat [23]. β-Pinene possesses a scent resembling fresh-cut grass and citrus aroma, and it has certain applications in the flavor and fragrance industry. 2-Methylbutanal is the embodiment of the intense spicy flavor in air-dried yak beef, which is likely produced by the metabolic activities of microorganisms during the natural air-drying and ripening process. Fernández et al. [24] has also demonstrated that microorganisms can enhance the formation of 2-methylbutanal in food. Other sulfur compounds primarily impart a pungent taste to cooked vegetables, onions, and garlic [25].

**Figure 3:**
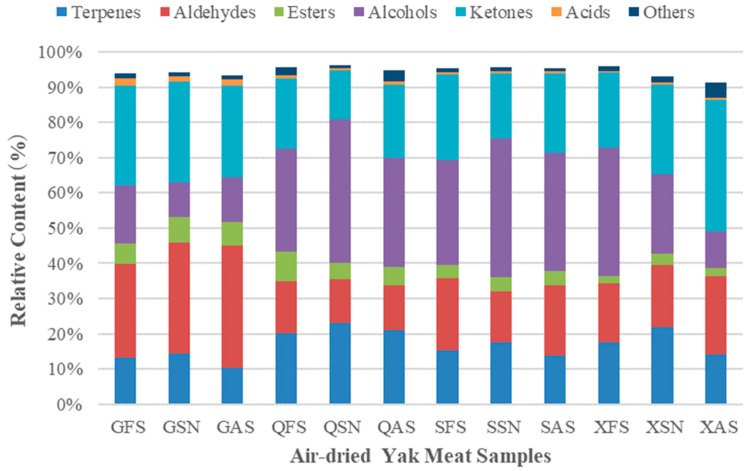
Relative content of volatile components in air-dried yak meat.

As depicted in Figure 3, the VOCs in 12 samples of air-dried yak beef are primarily composed of alcohols, ketones, and aldehydes. Among them, QSN has the highest content of terpenes (22.98%) and alcohols (40.59%), GAS has the highest aldehydes content (34.54%), QFS has the highest esters content (8.41%), XAS has the highest ketones content (37.50%), and GFS has the highest acids content (2.08%). The differences in these VOCs lead to variations in the taste among different air-dried yak beef samples. During the processing of AYM, the selection of appropriate spices not only introduces aromatic compounds to enhance the sensory attributes of the product but also elevates its nutritional quality and prolongs its shelf life. The results obtained from the analysis of ham, grass carp, braised chicken, and grilled lamb skewers exhibit similar trends [18,26,27,28].

### 3.3. VOC Fingerprints of AYM with Diverse Flavors from Four Regions

**Figure 4:**
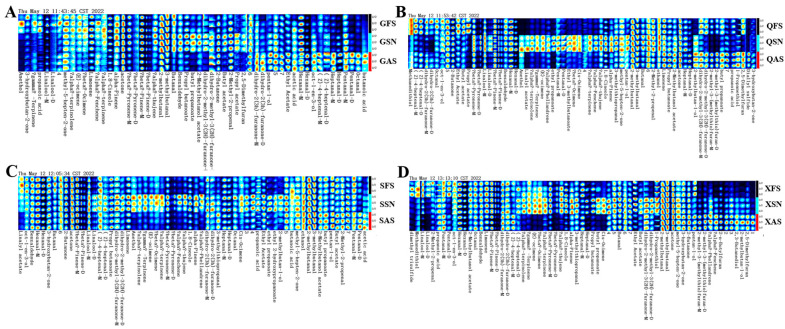
Relative content of volatile components in air-dried yak meat ((**A**–**D**) are Gansu, Qinghai, Tibet, and Sichuan regions, respectively).

From the topographic map and differential spectrum, one can observe the changing trends of VOCs in the sample. However, the qualitative analysis of VOCs necessitates the utilization of a fingerprint spectrum [29]. To more intuitively analyze the differences in VOCs of AYM from different regions and tastes, selected signal peaks with variations were utilized to construct a fingerprint plot of characteristic VOCs of AYM from four origins using the Gallery Plot plugin, as detailed in Figure 4A–D. Each row in the figure represents the signal peaks of a sample, and each column represents the signal peaks of the same VOC. Unknown compounds that have not been identified are labeled with numerical codes, and the color represents the intensity of the signal peaks in the AYM samples. A brighter color indicates a greater intensity. By comparing the dot intensities of the distribution of VOCs in various AYM samples, the changing trends in their VOCs can be determined.

In 12 types of AYM, the VOCs that are consistently present are 2-methylbutanal and 3-methylbutanal. Additionally, methyl-5-hepten-2-one is detected in AYM from the Gansu and Qinghai regions, while acetone is found in those from Sichuan and Tibet; the production processes of AYM in Sichuan and Tibet involve the incorporation of more chili peppers or other materials containing pungent VOCs, whereas in Gansu and Qinghai, the focus is predominantly on the inherent aroma of the meat, with minimal addition of ingredients that contribute strong flavors. Furthermore, the VOC content in AYM from Qinghai is more abundant, featuring a wider variety of compounds.

In Figure 4A, the primary VOCs of GFS are propanoic acid, γ-Terpinene, 3-hydroxybutan-2-one, and Anethol. The esters detected in GSN are significantly higher than those in the other two groups, including propyl butanoate, butyl propanoate, 2-methylbutanol acetate, butyl acetate, and propyl acetate, in addition to 2,5-dimethylfuran and benzaldehyde. On the other hand, GAS is primarily composed of ldehydes, such as octanal, hexanal, and pentanal-D. As shown in Figure 4B, the primary VOCs detected in QFS and QAS are 2,5-dimethylfuran, butyl propanoate, and dihydro-2-methyl-3(2H)-furanone. Additionally, QFS also contains propyl acetate and (Z)-4-heptenal. In contrast, QSN exhibits a more diverse array of VOCs, primarily terpenes (α-terpinolene, γ-terpinene, (E)-ocimene, β-ocimene, α-terpinene, α-fenchene, α-phellandrene, and cis-ocimene) and esters (linalyl acetate, ethyl 3-methylbutanoate, and ethyl propanoate).

The VOCs in SFS are primarily composed of linalyl acetate, while SAS is dominated by acetic acid and pentanal-D. SSN contains a variety of terpenes, including α-fenchene, α-thujene, and α-pinene, which are absent in QSN. In addition, SSN also contains ethyl propanoate, ethyl 2-hydroxypropanoate, 2-methylbutan-1-ol, and anethol (Figure 4C). Figure 4D shows that the VOCs in XFS include γ-terpinene, β-ocimene, (E)-ocimene, pentanal-D, linalool-M, methanedithiol, and dimethyl trisulfide. XSN, in addition to terpenes similar to those in QSN and SSN, also contains butyl propanoate. VOCs that are abundant in XAS are 2,5-dimethylfuran, 2-methylbutan-1-ol, α-fenchene, 2,3-butanediol, dihydro-2-methyl-3(2H)-furanone-M, and 2-methyl-3-(methylthio)furan-D.

Based on the above study, the amounts of VOCs present in SN samples from the four regions was the highest; this result indicates that during the production process, the SN sample incorporates a greater amount of spices during its production process than the other two flavors, aimed at enhancing its overall taste profile and catering to consumers with a preference for stronger flavors. GSN primarily consists of esters and alcohols, displaying differences in VOCs compared to QSN, SSN, and XSN. The remaining three regions were dominated by terpenes, containing the same VOCs, including α-terpinene, β-ocimene, (E)-ocimene, γ-terpinene, and α-terpinolene. These five compounds collectively exhibit a dominant pinewood aroma, coupled with a refreshing citrus or lemon scent. FS and AS from the four regions contained distinct VOCs, indicating that although they have a similar taste, differences in yak feeding methods, the drying processes for yak meat, and the ingredients and their dosages used in the production process vary among regions, leading to such differences. Therefore, we can distinguish dried yak meat with the same or different tastes from different regions based on the relative content of certain characteristic VOCs, achieving the identification of flavor characteristics in dried yak meat products.

### 3.4. Similarity Analysis of Flavor Components of AYM with Diverse Flavors in Four Regions

**Figure 5:**
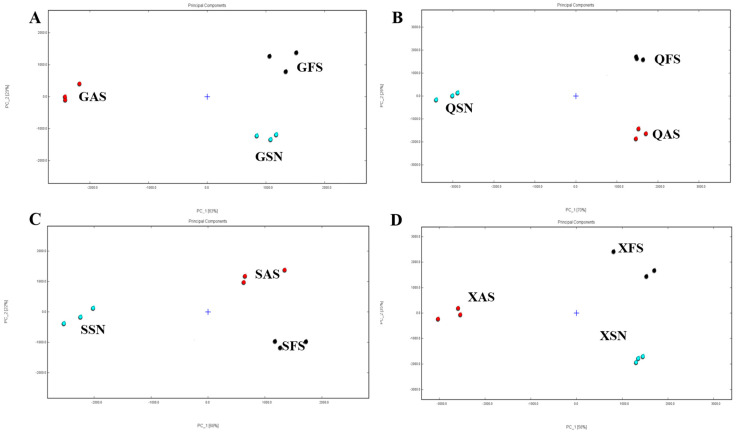
PCA of flavor compounds of air-dried yak meat with different flavors from four regions ((**A**–**D**) are Gansu, Qinghai, Tibet, and Sichuan regions, respectively).

PCA is the most commonly used method for feature extraction, which can evaluate the regularity and variability of VOCs in different samples by determining the contribution rate of two PC factors [30]. When the cumulative contribution rate reaches 60%, the PCA model can be selected as the separation model [31]. In this study, PCA was employed to analyze the variations in identified VOCs in AYM samples from four regions (Figure 5). The cumulative variance contribution rates of PC1 (63%) and PC2 (23%) in Gansu reached 86%, while those of PC1 (70%) and PC2 (26%) in Qinghai were 96%. Similarly, in Sichuan, the cumulative variance contribution rates of PC1 (68%) and PC2 (22%) were 90%, and in Tibet, the cumulative variance contribution rates of PC1 (58%) and PC2 (35%) totaled 93%. After dimensionality reduction, the cumulative contribution rates of the first two principal components remained above 85%, preserving relatively complete information and better representing the characteristic differences of the original variables. The aforementioned results demonstrate that significant differences exist in the flavor compounds among the three flavored AYM samples from four regions, allowing for clear differentiation of each flavor type.

**Figure 6:**
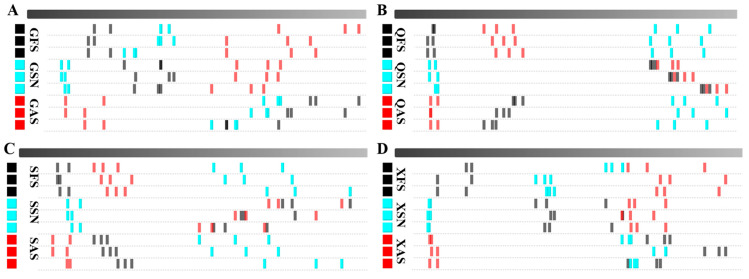
Euclidean distance plots of flavor compounds of air-dried yak meat with different flavors from four regions ((**A**–**D**) are Gansu, Qinghai, Tibet, and Sichuan regions, respectively).

Euclidean distance reflects the similarity between samples by measuring the distance between two vectors [32], and Euclidean distance plots can further distinguish samples that are not effectively separated via PCA. Figure 6A–D presents the Euclidean distance plot of air-dried yak beef samples with different flavors from four regions. The distance between samples is significantly greater than that between parallel samples, indicating that there are notable differences in the composition of VOCs among the air-dried yak beef with different flavors from the four regions. As observed in Figure 6A,D, the distances between the FS and AS from Gansu and Tibet are relatively far, indicating a low similarity. In contrast, the distances between Qinghai and Sichuan are closer, suggesting a higher similarity, while they are further apart from the SN, exhibiting distinct differences (Figure 6B,C). These results are consistent with the PCA findings.

In conclusion, GAS and XAS exhibit high degrees of difference and low similarity compared to the other two flavors in their respective regions. GFS and GSN, as well as XFS and XSN, display slight differences among themselves. QSN and SSN are the most distinctive flavors in Qinghai and Sichuan, respectively. Furthermore, the similarity between QFS and QAS is higher than that between SFS and SAS.

### 3.5. PLS-DA and Model Assessment Analysis and Screening of Differential Volatile Components

PLS-DA is frequently utilized to handle classification and discrimination problems [33]. To identify VOCs exhibiting differential volatility in AYM and to select samples showing significant flavor distinctions, we constructed a PLS-DA model. In evaluating the model’s performance, Q2 (predictive index) is used to measure the predictive ability of the model, while R2X and R2Y (goodness-of-fit indices) are employed to assess the goodness of fit and reliability of the model. Values of R2 and Q2 exceeding 0.5 indicate an acceptable model fit [34]. These parameters range from 0 to 1, and the closer the value is to 1, the stronger the predictability or interpretability of the model. Additionally, the reliability of the PLS-DA model was verified through permutation testing. After 200 cross-validations, if the regression line of model Q intersects the horizontal axis with a negative intercept, and all permutation test R2 and Q2 values are lower than the initial values, it can be proven that the model is not overfitted [35]. In calculating the contribution of each VOC to classification, we adopted Variable Importance in Projection (VIP) as an indicator. The VIP value is typically used to describe the magnitude of a variable’s contribution to the model, where variables with a VIP value greater than 1 are considered to have the most significant impact on the model [36].

#### 3.5.1. Analysis and Screening of PLS-DA and Model Evaluation of Differences in Volatile Components of 12 Samples of AYM

**Figure 7:**
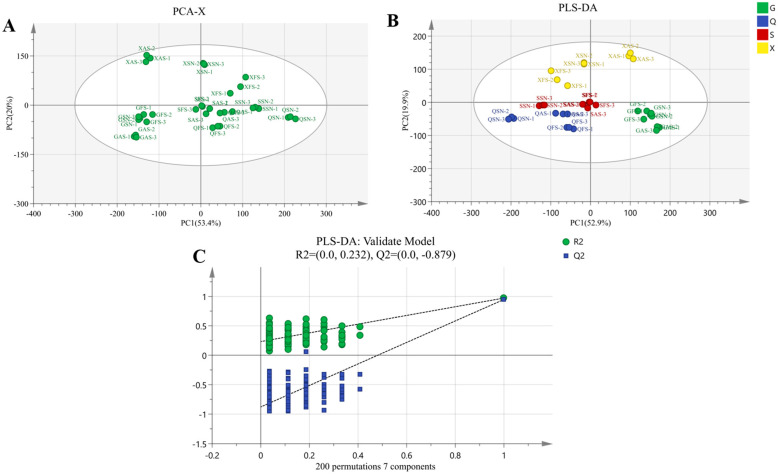
(**A**) Scatter plot of PCA of 12 air-dried yak meat samples. (**B**) Plot of volatile components scores of 12 air-dried yak meat samples categorized by Gansu (G), Qinghai (Q), Sichuan (S), and Tibet (X). (**C**) Substitution test plot of 12 air-dried yak meats.

The sum of contributions of the first and second principal components of the overall PCA score plot for 12 kinds of AYM was 73.4%, and they were well separated in the plot (Figure 7A). Samples XFS and XSN were primarily located in the first quadrant, while sample XAS was mainly in the second quadrant. Samples GFS, GSN, and GAS were mainly located in the third quadrant, with partial overlap between GFS and GSN. QFS, QSN, QAS, SSN, and SAS were all located in the fourth quadrant. SFS was positioned at the center of the circle. There were significant variations in the flavor of AYM from the four origins.

As shown in Figure 7B, the AYM samples from Tibet were primarily distributed in the first and second quadrants, while those from Qinghai were mainly in the third quadrant. Samples from Gansu were mostly in the fourth quadrant, and the Sichuan samples, except for the spicy flavor distributed in the third quadrant, were clustered at the center of the circle. In this study, the values of R2X, R2Y, and Q2 were 0.957, 0.97, and 0.94, respectively. The model exhibited good fitting, and its predictability was acceptable (Figure 7C). The PLS-DA model more accurately identified the VOCs of the 12 kinds of AYM, while effectively eliminating non-relevant variable substances, thus resulting in differences from the PCA results. The degree of differentiation among samples was improved, making it more convenient for observation and analysis.

**Figure 8:**
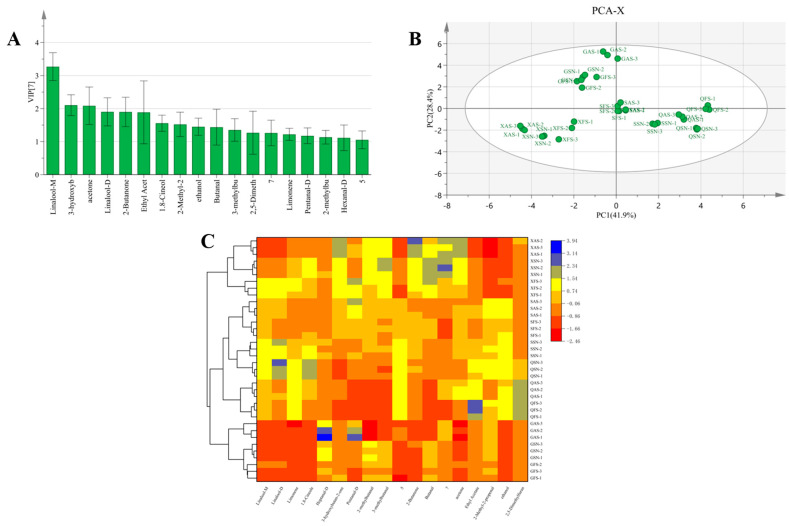
Screening of differential volatile components in 12 air-dried yak meat samples ((**A**) VOCs with VIP values > 1; (**B**) PCA scoring plot; (**C**) clustered thermogram).

As shown in Figure 8A, VIP values were utilized to screen VOCs in 12 AYM samples, and 17 VOCs with VIP values greater than 1 were identified, including linalool, limonene, 1.8-cineole, heptanal-D, 3-hydroxybutan-2-one, pentanal-D, 2-methylbutanal, 3-methylbutanal, 2-butanone, butanal, acetone, ethyl acetate, 2-methyl-2-propenal, ethanol, and 2,5-dimethylfuran. PCA and cluster analysis were further applied to analyze these 17 VOCs. The variations could still be explained by the contributions of PC1 and PC2, which were 41.9% and 28.4%, respectively (Figure 8B). The clustering heatmap (Figure 8C) also demonstrated that the 17 VOCs effectively discriminated the differences among the samples. Therefore, the identification of different flavors of AYM from different regions can be achieved through the screening of volatile compound markers, combined with principal component analysis and cluster analysis.

#### 3.5.2. PLS-DA and Model Evaluation Analysis and Screening of Differential VOCs of AYM with FS

**Figure 9:**
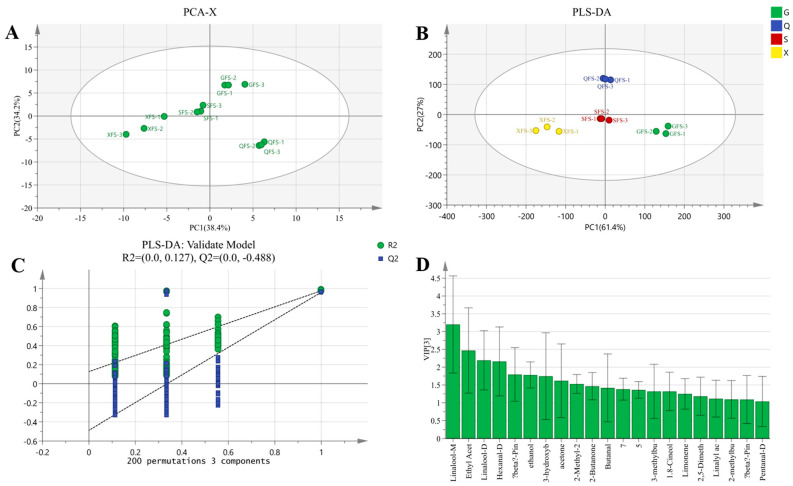
(**A**) PCA scatter plot of air-dried yak meat with FS flavoring. (**B**) Volatile component score plot of four air-dried yak meat samples with FS flavoring classified according to Gansu (G), Qinghai (Q), Sichuan (S), and Tibet (X). (**C**) Substitution test plot of air-dried yak meat with FS flavoring. (**D**) VOCs in air-dried yak meat with FS flavoring with VIP value greater than 1.

Figure 9A displays the PCA score plot of FS from four regions, with the cumulative variance contribution of PC1 (38.4%) and PC2 (34.2%) totaling 72.6%. Based on this, a PLS-DA model for FS was established (Figure 9B), wherein QFS samples are mainly concentrated in the center of the first and second quadrants, XFS samples are entirely located in the third quadrant, GFS samples are entirely distributed in the fourth quadrant, and among the three parallel samples of SFS, most belong to the third quadrant and are somewhat distant from the XFS samples. The samples cluster closely together and are far from other FS samples, enabling a clear distinction of FS from the four regions. In the alternative test plot of FS (Figure 9C), the regression line of model Q intersects with the *x*-axis at a negative intercept. Additionally, R2X, R2Y, and Q2 are 0.957, 0.987, and 0.979, respectively, all approaching 1, indicating the reliability of the model.

To further investigate specific differential VOCs, VOCs with VIP values greater than 1 were screened from all detected VOCs (Figure 9D). A total of 19 VOCs were identified, including known VOCs such as linalool, limonene, β-pinene, 1.8-cineole, hexanal-D, 3-hydroxybutan-2-one, pentanal-D, 2-methylbutanal, 3-methylbutanal, 2-butanone, butanal, acetone, ethyl acetate, 2-methyl-2-propenal, ethanol, and 2,5-dimethylfuran. Apart from the common VOCs shared by all AYM samples, the characteristic flavor compound of FS is linalyl acetate, which possesses a mild grassy aroma. However, the VOCs that contribute the most significantly to the taste of FS are ethyl acetate, hexanal-D, and β-pinene, resembling the key aroma compounds identified in the VOCs of Yanbian-style flavorful beef stew by Li et al. [37].

#### 3.5.3. PLS-DA and Model Evaluation Analysis and Screening of Differential VOCs of AYM with SN

**Figure 10:**
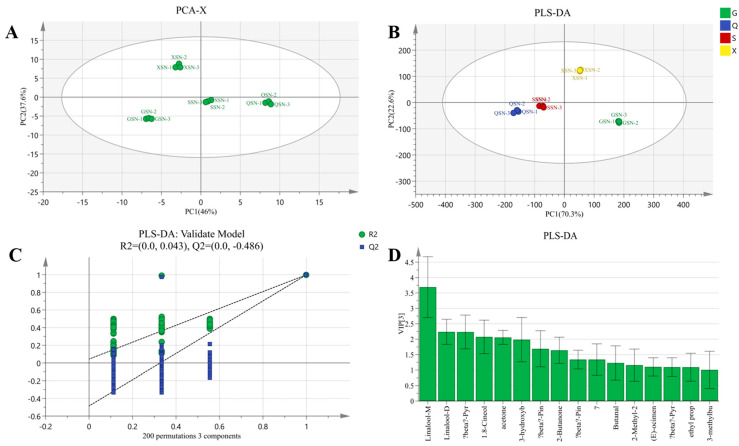
(**A**) PCA scatter plot of air-dried yak meat with SN flavoring. (**B**) Volatile component score plot of four air-dried yak meat samples with SN flavoring classified according to Gansu (G), Qinghai (Q), Sichuan (S), and Tibet (X). (**C**) Substitution test plot of air-dried yak meat with SN flavoring. (**D**) VOCs in air-dried yak meat with SN flavoring with VIP value greater than 1.

Based on Figure 10A, PCA score plots of SN samples from four regions were obtained, with cumulative variance contribution rates of PC1 (46%) and PC2 (37.6%) reaching 83.6%. In the PCA plot, SN samples with the same flavor from the four regions can be clearly distinguished. For the PLS-DA model (Figure 10B), the R2X, R2Y, and Q2 values were 0.957, 0.987, and 0.979, respectively, indicating a robust and reliable model (Figure 10C). In this model, XSN samples primarily clustered in the first quadrant, while QSN and SSN samples grouped separately in the second quadrant. GSN samples were entirely located in the fourth quadrant. The parallel samples of the same specimen are closely clustered, while maintaining a distinct distance from other SN samples. This demonstrates that the use of multivariate analysis can effectively distinguish the AYM of the SN group based on their VOCs.

A total of 13 VOCs with VIP values greater than 1 were identified in SN samples through differential screening (Figure 10D). The known VOCs include linalool, (E)-ocimene, β-pyronene, β-pinene, 1.8-cineole, 3-hydroxybutan-2-one, 3-methylbutanal, 2-butanone, butanal, acetone, 2-methyl-2-propenal, and ethyl propanoate. After excluding the VOCs commonly found in air-dried yak beef, the characteristic flavor compounds in SN samples are (E)-ocimene and ethyl propanoate. (E)-ocimene possesses a fresh, herbal, and natural aroma, while ethyl propanoate can impart a pineapple-like fragrance to foods. β-pyronene, 1.8-vineole, acetone, and 3-hydroxybutan-2-one are VOCs that significantly contribute to the FS samples. Notably, 1.8-cineole, a component of dried ginger extract, exhibits a fresh, intense, camphor-like, and faintly minty aroma [38]. Its presence in SN AYM is likely due to the addition of ginger slices or mint during the manufacturing process. Additionally, other ketones function as seasoning ingredients that impart a spicy and pungent flavor.

#### 3.5.4. PLS-DA and Model Evaluation Analysis and Screening of Differential VOCs of AYM with AS

**Figure 11:**
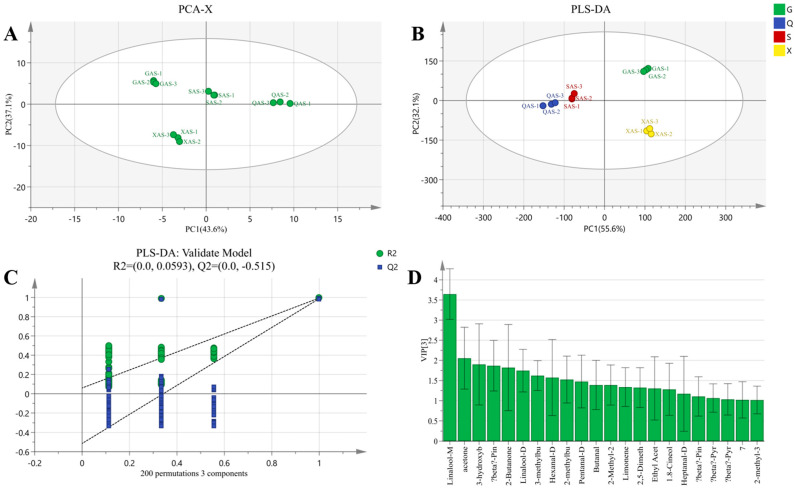
(**A**) PCA scatter plot of air-dried yak meat with AS flavoring. (**B**) Volatile component score plot of four air-dried yak meat samples with AS flavoring classified according to Gansu (G), Qinghai (Q), Sichuan (S), and Tibet (X). (**C**) Substitution test plot of air-dried yak meat with AS flavoring. (**D**) VOCs in air-dried yak meat with AS flavoring with VIP value greater than 1.

The PCA score plot of AS samples from four regions is presented in Figure 11A, with cumulative variance contributions of PC1 (43.6%) and PC2 (37.1%) totaling 80.7%. PCA can effectively distinguish AS samples from the four regions. After establishing the PLS-DA model, slight differences from the PCA results are observed (Figure 11B). Specifically, GAS samples are mainly clustered in the first quadrant, while SAS and QAS samples are distributed in the second and third quadrants, respectively, and are close to each other. In contrast, all XAS samples are located in the fourth quadrant. Figure 11C displays R2 and Q2 intercept values of (0, 0.059) and (0, −0.52). All Q2 points are below the original Q2 point on the rightmost side, and the Q2 regression line intersects the vertical axis at a value less than 0, indicating that the PLS-DA model is robust, reliable, and without overfitting. These results demonstrate that the use of multivariate analysis can effectively differentiate AS air-dried yak meat based on their VOCs.

A total of 19 VOCs with VIP values greater than 1 in AS samples were screened through differential analysis (Figure 11D). Among them, the known VOCs include linalool, limonene, β-pyronene, β-pinene, 1.8-cineole, 2-methyl-3-(methylthio) furan-D, heptanal-D, hexanal-D, 3-hydroxybutan-2-one, pentanal-D, 2-methylbutanal, 3-methylbutanal, 2-butanone, butanal, acetone, ethyl acetate, 2-methyl-2-propenal, and 2,5-dimethylfuran. After excluding VOCs commonly present in AYM, 2-methyl-3-(methylthio)furan-D and hexanal-D were identified as the characteristic flavor compounds in AS samples. 2-methyl-3-(methylthio)furan-D primarily exhibits meaty and onion–garlic aroma, while hexanal-D possesses a grassy, fruity, and fatty aroma. The VOCs that contribute significantly to the aroma components of AS are acetone, 3-hydroxybutan-2-one, β-pinene, and 2-butanone, exhibiting similar compositions with FS and SN samples. Consistent with the findings of Huang et al., VOCs such as 3-hydroxybutan-2-one and 2-butanone undergo oxidation and gradually increase during the natural drying process [39].

In summary, based on the evaluation of the PLS-DA model and the screening results of differential volatile components, it is concluded that VOCs commonly found in AYM include aldehydes (3-methylbutanal, butanal, and 2-methyl-2-propenal), ketones (3-hydroxybutan-2-one, 2-butanone, and acetone), and alcohols (linalool and 1.8-cineole). Aldehydes and ketones possess a strong and pungent odor, while alcohols exhibit a refreshing citrus and floral aroma. Compared with FS and AS, the number of volatile differential substances screened from SN samples is the least, while that from AS samples is the most. These results suggest that multivariate analysis can be applied to distinguish different samples and screen for characteristic VOCs in AYM from GC–IMS data.

## 4. Conclusions

In this study, GC–IMS was utilized to comprehensively analyze and compare the VOCs in AYM samples with three flavors (FS, SN, and AS) from four regions of the Qinghai-Tibet Plateau (Gansu, Qinghai, Sichuan, and Tibet). A total of 58 VOCs were detected, including 13 terpenes, 11 aldehydes, 11 esters, 9 alcohols, 5 ketones, 3 acids, and 6 other compounds. Among them, 2-methylbutanal, 3-methylbutanal, butanal, 2-methyl-2-propenal, 3-hydroxybutan-2-one, 2-butanone, acetone, alcohols, linalool, and 1.8-cineole were the major VOCs in the AYM. Specifically, linalyl acetate was identified as the characteristic volatile VOC in FS air-dried yak meat, while (E)-ocimene and ethyl propanoate were the distinguishing VOCs in SN air-dried yak meat, and 2-methyl-3-(methylthio)furan-D and hexanal-D were unique to AS. Furthermore, the research results demonstrate that not only do the AYM samples with the same flavor from different regions exhibit relatively independent and clearly distinguishable patterns in PCA and PLS-DA plots, but also the 12 samples of AYM with different flavors and originating from various regions do so as well. Cluster heatmap analysis further confirmed that the aroma characteristics of the 12 samples could be distinguished based on VOCs. In summary, GC–IMS combined with multivariate analysis provides a rapid and reliable method for flavor profiling, which aids in better distinguishing the VOC characteristics of AYM and identifying AYM with different flavors from various regions. On the one hand, the flavor of AYM can be improved in the food industry to better align with public preferences, thereby boosting the sales of yak meat and ultimately generating economic benefits for the Qinghai-Tibet Plateau region. However, on the other hand, this study exhibits certain limitations. Specifically, it solely focused on the flavor aspects of AYM, neglecting further exploration into its food quality. In the future, quality inspections and authenticity identifications can be conducted on the obtained AYM samples to ensure that consumers purchase high-quality and genuine yak meat.

## Figures and Tables

**Table 1:** Sample information.

Number	Sample Code	Instruction	Producers
1	GFS	Gansu Five-Spice Flavor	Gansu Shanlima Foods Co. Gansu, China.
2	GSN	Gansu Spicy and Numbing Flavor	
3	GAS	Gansu Aromatic and Spicy Flavor	
4	QFS	Qinghai Five-Spice Flavor	Qinghai Wenyou Food Co. Qinghai, China.
5	QSN	Qinghai Spicy and Numbing Flavor	
6	QAS	Qinghai Aromatic and Spicy Flavor	
7	SFS	Sichuan Five-Spice Flavor	Ganzi Luding County Minqiao Story Agricultural Products Development Co. Sichuan, China.
8	SSN	Sichuan Spicy and Numbing Flavor	
9	SAS	Sichuan Aromatic and Spicy Flavor	
10	XFS	Xizang Five-Spice Flavor	Tibet Qisheng Native Specialty Products Co. Lhasa, China.
11	XSN	Xizang Spicy and Numbing Flavor	
12	XAS	Xizang Aromatic and Spicy Flavor	

**Table 2:** Results of qualitative analysis of volatile flavor compounds in air-dried yak meat.

Number	Compound	CAS#	RI	Rt [s]	Dt [a.u.]	Aroma Characteristics
	**Terpenes (** **13** **)**					
1	α-terpinolene	586-62-9	1084	471	1.22	pine
2	γ -Terpinene	99-85-4	1063	441	1.22	pungent
3	(E)-ocimene(E)	3779-61-1	1052	426	1.22	freshness
4	β-Ocimene	13877-91-3	1044	415	1.21	herbal
5	Limonene	138-86-3	1023	384	1.22	citrus
6	α-terpinene	99-86-5	1013	370	1.22	pine
7	β-Pyronene-D	514-96-5	993	343	1.71	woody, herbaceous
	β-Pyronene-M	514-96-5	993	343	1.22	
8	β-Pinene-D	127-91-3	972	325	1.64	herbal, resinous
	β-Pinene-M	127-91-3	972	325	1.22	
9	α-Fenchene	471-84-1	944	301	1.22	woody, herbaceous
10	α-thujene	2867-05-2	922	282	1.22	pine, herbaceous
11	α-Pinene	80-56-8	930	289	1.22	pine, woody, and herbaceous
12	α-Phellandrene	99-83-2	1006	360	1.22	black pepper, mint
13	Cis-Ocimene	3338-55-4	1034	400	1.22	basil
	**Aldehydes (** **11** **)**					
1	Benzaldehyde	100-52-7	958	313	1.15	
2	3-methylthiopropanal	3268-49-3	905	268	1.09	
3	(Z)-4-heptenal-D	6728-31-0	897	261	1.62	
	(Z)-4-heptenal-M	6728-31-0	896	260	1.15	
4	Heptanal-M	111-71-7	900	264	1.33	fatty
	Heptanal-D	111-71-7	897	2611	1.69	
5	Octanal	124-13-0	1005	358	1.40	
6	Hexanal-M	66-25-1	793	204	1.26	grassy
	Hexanal-D	66-25-1	793	204	1.56	
7	Pentanal-D	110-62-3	695	163	1.421	grassy, vegetable
	Pentanal-M	110-62-3	694.	163	1.19	
8	2-methylbutanal	96-17-3	665	154	1.40	spicy
9	3-methylbutanal	590-86-3	642	148	1.41	nuts
10	Butanal	123-72-8	545	122	1.28	herbaceous, spicy
11	2-Methyl-2-propenal	78-85-3	553	124	1.22	
	**Esters (** **11** **)**					
1	Linalyl acetate	115-95-7	1396	920	1.22	floral, fruity
2	dihydro-2(3h)-furanone-M	96-48-0	917	278	1.08	
	dihydro-2(3h)-furanone-D	96-48-0	916	277	1.30	
3	Propyl butanoate	105-66-8	895	259	1.26	
4	butyl propanoate	590-01-2	907	270	1.29	
5	2-Methylbutanol acetate	624-41-9	879	250	1.32	
6	Ethyl Acetate	141-78-6	604	137	1.34	fruity, sweet
7	Butyl acetate	123-86-4	805	210	1.24	
8	Propyl acetate	109-60-4	707	168	1.48	
9	Ethyl 3-methylbutanoate	108-64-5	846	232	1.25	
10	ethyl propanoate	105-37-3	704	167	1.45	
11	ethyl 2-hydroxypropanoate	97-64-3	808	212	1.14	
	**Alcohols (** **9** **)**					
1	Linalool-M	78-70-6	1108	507	1.22	floral, citrus
	Linalool-D	78-70-6	1106	503	1.70	
2	1.8-Cineole	470-82-6	1025	387	1.29	camphorated, coolness
3	oct-1-en-3-ol	3391-86-4	981	333	1.16	
4	2,3-Butanediol	513-85-9	780	197	1.37	
5	ethanol	64-17-5	451	96	1.13	
6	1-Propanethiol	107-03-9	625	143	1.37	
7	2-methylbutan-1-ol	137-32-6	742	182	1.47	
8	Methanedithiol	6725-64-0	736	179	1.04	
9	pentan-1-ol-	71-41-0	761	190	1.25	
	**Ketones (** **5** **)**					
1	methyl-5-hepten-2-one	110-93-0	992	342	1.18	
2	dihydro-2-methyl-3(2H)-furanone-D	3188-00-9	802	208	1.42	nuts
	dihydro-2-methyl-3(2H)-furanone-M	3188-00-9	801	208	1.07	
3	3-hydroxybutan-2-one	513-86-0	709	169	1.33	creamy, fatty
4	2-Butanone	78-93-3	579	131	1.25	
5	acetone	67-64-1	490	107	1.12	spicy
	**Acids (3)**					
1	propanoic acid	79-09-4	689	160	1.11	
2	acetic acid	64-19-7	580	131	1.16	
3	butanoic acid	107-92-6	813	214	1.16	
	**Others (6)**					
1	2-methyl-3-(methylthio)furan-M	63012-97-5	947	303	1.11	meaty, garlic odor
	2-methyl-3-(methylthio)furan-D	63012-97-5	948	304	1.15	
2	Anethol	104461	1453	1002	1.21	aniseed
3	2-n-Butylfuran	4466244	875	248	1.18	
4	dimethyl trisulfide	3658808	958	313	1.30	
5	Diallyl sulfide	592881	851	235	1.12	
6	2,5-Dimethylfuran	625865	694	162	1.03	caramel, nuts

## Data Availability

The original contributions presented in the study are included in the article, further inquiries can be directed to the corresponding authors.
